# 5-Methyl-3-[1-(2-pyridylmeth­yl)-1*H*-benzimidazol-2-ylmeth­yl]isoxazole

**DOI:** 10.1107/S1600536809040100

**Published:** 2009-10-10

**Authors:** Mohamadou Lamine Doumbia, Rachid Bouhfid, El Mokhtar Essassi, Lahcen El Ammari

**Affiliations:** a Laboratoire de Chimie Organique Hétérocyclique, Pôle de compétences, Pharmacochimie, Av Ibn Battouta, BP 1014, Faculté des Sciences, Université Mohammed V-Agdal, Rabat, Morocco; b INANOTECH, Rabat, Morocco; c Laboratoire de Chimie du Solide Appliquée, Faculté des Sciences, Université Mohammed V-Agdal, Avenue Ibn Battouta, BP. 1014, Rabat, Morocco

## Abstract

The title compound, C_18_H_16_N_4_O, is built up from fused six- and five-membered rings linked to a five-membered isoxazole ring and to a six-membered pyridine ring through a CH_2_ group. The fused-ring system is essentially planar, with a maximum deviation of 0.019 (1) Å. It forms inter­planar angles of 70.03 (7)° with the isoxazole ring and 81.68 (7)° with the pyridine ring; the two latter rings are also planar, the maximum deviations from the mean planes being 0.0028 (15) and 0.0047 (12) Å, respectively. In the crystal, weak inter­molecular non-classical C—H⋯N hydrogen bonds link the mol­ecules, forming a zigzag-like chain parallel to the *b* axis. A weak intra­molecular C—H⋯N hydrogen bond may help to define the conformation of the mol­ecule.

## Related literature

Isoxazoles and their derivatives are key inter­mediates for the preparation of products which mimics natural compounds, see: Baraldi *et al.* (1987 ▶). For their biological activity, see: Boros *et al.* (2006 ▶); Desai & Desai (2006 ▶); Eddington *et al.* (2002 ▶); Kang *et al.* (2000 ▶); Ko *et al.* (1998 ▶); Lee & Kim (2002 ▶); Sbai *et al.* (2003 ▶).
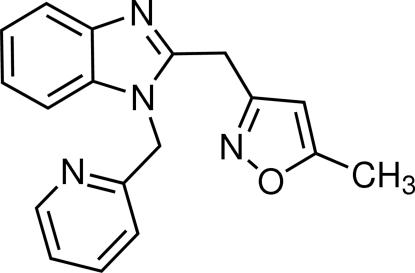


## Experimental

### 

#### Crystal data

C_18_H_16_N_4_O
*M* *_r_* = 304.35
Monoclinic, 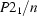

*a* = 11.0761 (2) Å
*b* = 8.6535 (1) Å
*c* = 16.5920 (3) Å
β = 103.136 (1)°
*V* = 1548.68 (4) Å^3^
*Z* = 4
Mo *K*α radiation
μ = 0.09 mm^−1^
*T* = 298 K
0.28 × 0.16 × 0.06 mm

#### Data collection

Bruker X8  Kappa APEX II diffractometer
Absorption correction: none
29777 measured reflections
3567 independent reflections
2460 reflections with *I* > 2σ(*I*)
*R*_int_ = 0.047

#### Refinement

*R*[*F* ^2^ > 2σ(*F* ^2^)] = 0.040
*wR*(*F* ^2^) = 0.105
*S* = 1.01
3567 reflections
214 parameters
H-atom parameters constrained
Δρ_max_ = 0.15 e Å^−3^
Δρ_min_ = −0.14 e Å^−3^

### 

Data collection: *APEX2* (Bruker, 2005 ▶); cell refinement: *SAINT* (Bruker, 2005 ▶); data reduction: *SAINT*; program(s) used to solve structure: *SHELXS97* (Sheldrick, 2008 ▶); program(s) used to refine structure: *SHELXL97* (Sheldrick, 2008 ▶); molecular graphics: *ORTEP-3 for Windows* (Farrugia, 1997 ▶) and *PLATON* (Spek, 2009 ▶); software used to prepare material for publication: *WinGX* (Farrugia, 1999 ▶).

## Supplementary Material

Crystal structure: contains datablocks I, global. DOI: 10.1107/S1600536809040100/dn2494sup1.cif

Structure factors: contains datablocks I. DOI: 10.1107/S1600536809040100/dn2494Isup2.hkl

Additional supplementary materials:  crystallographic information; 3D view; checkCIF report

## Figures and Tables

**Table 1:** Hydrogen-bond geometry (Å, °)

*D*—H⋯*A*	*D*—H	H⋯*A*	*D*⋯*A*	*D*—H⋯*A*
C2—H2⋯N4^i^	0.93	2.52	3.385 (2)	155
C12—H12*B*⋯N1	0.97	2.60	3.479 (2)	151

Symmetry code: (i) 
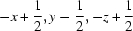
.

**Fig. 1.**
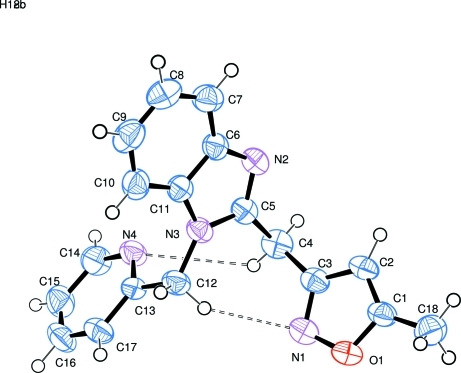
: Molecular view with the atom labeling scheme. Displacement ellipsoids are drawn at the 50% probability level. H atoms are represented as small spheres of arbitrary radii. H bonds are shown as dashed lines.

**Fig. 2.**
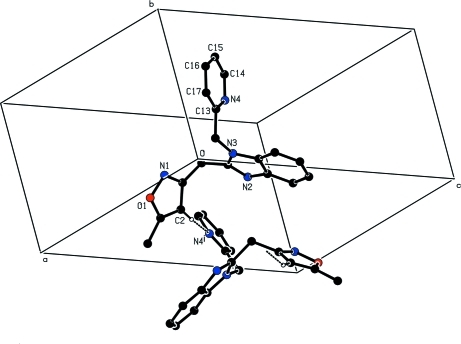
: Partial packing view showing the formation of a chain through C—H···N hydrogen bond shown as dashed lines. H atoms not involved in hydrogen bondings have been omitted for clarity. [Symmetry code: (i) 1/2 - x, y - 1/2, 1/2 - z]

## supplementary crystallographic information

### Comment

Isoxazoles and their derivatives are key intermediates for the preparation of products which mimics natural compounds (Baraldi *et al.*, 1987). They have long been targeted in synthetic investigation for their known biological activities and pharmacological properties such as antiviral (Lee & Kim, 2002), anticonvulsant (Eddington *et al.*, 2002), anti-inflammatory (Ko *et al.*, 1998), and anti-bacterial activity (Kang *et al.*, 2000). Also, varied pharmacological and chelating properties are associated with benzimidazole derivatives and pyridine (Sbai *et al.*, 2003, Desai & Desai, 2006, Boros *et al.* 2006). Thus it is expected that the association of benzimidazole and pyridine moiety with the isoxazole system would affect significantly the biological and complexing properties.

The molecule is built up from fused six and five-membered rings linked together to a five-membered isoxazole ring and to six-membered pyridine ring through a CH_2_ chain (Fig. 1). The fused ring system is essentially planar, with maximum deviation of -0.008 (2) and -0.019 (1) Å for atoms C4 and C12 respectively. It forms interplanar angles of 70.03 (7)° with the isoxazole ring and 81.68 (7)°t with the pyridine ring. These two last rings are also planar with maximum deviation from the mean planes being 0.0028 (15) at C1 and 0.0047 (12) at N4 for the isoxazole and the pyridine ring respectively.

There is a a weak intermolecular non classical C—H···N hydrogen bond linking the molecules to form a chain parallel to the (Table 1, Fig. 1). Weak intramolecular hydrogen bonds C—H···N may be responsible for the conformation of the molecule (Fig. 1, Table 1).

### Experimental

To a solution of 3-((1*H*-benzimidazol-2-yl)methyl)-5-methylisoxazole (0.85 g, 4 mmol) in DMF (20 ml) was added (0.60 g, 4.4 mmol) of K2CO3. The mixture was stirred for 10 min at rt, and then 2-(chloromethyl)pyridine hydrochloride (0.72 g, 4.4 mmol) was added to the mixture. The reaction mixture was stirred at room temperature for 24 h. whereupon a white solid was deposited. The solid was filtered off, and the filtrate was concentrated under reduced pressure. The residue was subjected to a column chromatography (silica gel, hexane/ethyl acetate, 7:3, *v*/*v*) to give 5-methyl-3-((1-(pyridin-2-ylmethyl)-1*H*-benzimidazol-2-yl)methyl)isoxazole as a white crystal in 65% yield (0.79 g).

### Refinement

All H atoms were fixed geometrically and treated as riding with C—H = 0.96 Å (methyl), 0.97 Å (methylene) or 0.93 Å (aromatic) with *U*_iso_(H) = 1.2*U*_eq_(aromatic, methylene) or *U*_iso_(H) = 1.5*U*_eq_(methyl).

### Crystal data

**Table:**

C_18_H_16_N_4_O	*F*(000) = 640
*M**_r_* = 304.35	*D*_x_ = 1.305 Mg m^−^^3^
Monoclinic, *P*2_1_/*n*	Mo *K*α radiation, λ = 0.71073 Å
Hall symbol: -P 2yn	Cell parameters from 3567 reflections
*a* = 11.0761 (2) Å	θ = 2.5–27.5°
*b* = 8.6535 (1) Å	µ = 0.09 mm^−^^1^
*c* = 16.5920 (3) Å	*T* = 298 K
β = 103.136 (1)°	Block, white
*V* = 1548.68 (4) Å^3^	0.28 × 0.16 × 0.06 mm
*Z* = 4	

### Data collection

**Table:**

Bruker X8 APEX Diffractometer	2460 reflections with *I* > 2σ(*I*)
Radiation source: fine-focus sealed tube	*R*_int_ = 0.047
graphite	θ_max_ = 27.5°, θ_min_ = 2.5°
φ and ω scans	*h* = −14→14
29777 measured reflections	*k* = −11→11
3567 independent reflections	*l* = −21→17

### Refinement

**Table:**

Refinement on *F*^2^	Secondary atom site location: difference Fourier map
Least-squares matrix: full	Hydrogen site location: inferred from neighbouring sites
*R*[*F*^2^ > 2σ(*F*^2^)] = 0.040	H-atom parameters constrained
*wR*(*F*^2^) = 0.105	*w* = 1/[σ^2^(*F*_o_^2^) + (0.0445*P*)^2^ + 0.2578*P*] where *P* = (*F*_o_^2^ + 2*F*_c_^2^)/3
*S* = 1.01	(Δ/σ)_max_ < 0.001
3567 reflections	Δρ_max_ = 0.15 e Å^−^^3^
214 parameters	Δρ_min_ = −0.14 e Å^−^^3^
0 restraints	Extinction correction: *SHELXL97* (Sheldrick, 2008), Fc^*^=kFc[1+0.001xFc^2^λ^3^/sin(2θ)]^-1/4^
Primary atom site location: structure-invariant direct methods	Extinction coefficient: 0.0099 (13)

### Special details

**Table:**

Geometry. All e.s.d.'s (except the e.s.d. in the dihedral angle between two l.s. planes) are estimated using the full covariance matrix. The cell e.s.d.'s are taken into account individually in the estimation of e.s.d.'s in distances, angles and torsion angles; correlations between e.s.d.'s in cell parameters are only used when they are defined by crystal symmetry. An approximate (isotropic) treatment of cell e.s.d.'s is used for estimating e.s.d.'s involving l.s. planes.
Refinement. Refinement of *F*^2^ against ALL reflections. The weighted *R*-factor *wR* and goodness of fit *S* are based on *F*^2^, conventional *R*-factors *R* are based on *F*, with *F* set to zero for negative *F*^2^. The threshold expression of *F*^2^ > σ(*F*^2^) is used only for calculating *R*-factors(gt) *etc*. and is not relevant to the choice of reflections for refinement. *R*-factors based on *F*^2^ are statistically about twice as large as those based on *F*, and *R*- factors based on ALL data will be even larger.

### Fractional atomic coordinates and isotropic or equivalent isotropic displacement parameters (Å^2^)

**Table:**

	*x*	*y*	*z*	*U*_iso_*/*U*_eq_	
O1	0.35108 (10)	−0.03671 (13)	0.02360 (6)	0.0584 (3)	
N1	0.28238 (12)	0.07631 (17)	0.05503 (8)	0.0587 (4)	
N2	0.14275 (11)	0.00694 (14)	0.28340 (7)	0.0462 (3)	
N3	0.02122 (11)	0.07134 (13)	0.16088 (7)	0.0421 (3)	
N4	−0.03858 (11)	0.38647 (14)	0.14258 (7)	0.0472 (3)	
C1	0.41164 (13)	−0.12534 (17)	0.08719 (9)	0.0461 (4)	
C2	0.38459 (14)	−0.07581 (17)	0.15741 (9)	0.0467 (4)	
H2	0.4130	−0.1163	0.2102	0.056*	
C3	0.30411 (13)	0.05044 (16)	0.13448 (9)	0.0438 (3)	
C4	0.24511 (14)	0.15139 (17)	0.18861 (9)	0.0519 (4)	
H4B	0.3067	0.1774	0.2384	0.061 (5)*	
H4A	0.2178	0.2469	0.1595	0.067 (5)*	
C5	0.13705 (13)	0.07608 (15)	0.21254 (8)	0.0414 (3)	
C6	0.02351 (13)	−0.04756 (15)	0.27876 (8)	0.0428 (3)	
C7	−0.02328 (16)	−0.13135 (18)	0.33632 (10)	0.0559 (4)	
H7	0.0275	−0.1608	0.3866	0.067*	
C8	−0.14703 (17)	−0.1693 (2)	0.31653 (11)	0.0655 (5)	
H8	−0.1803	−0.2249	0.3543	0.079*	
C9	−0.22337 (16)	−0.1265 (2)	0.24140 (11)	0.0657 (5)	
H9	−0.3067	−0.1538	0.2302	0.079*	
C10	−0.17938 (14)	−0.04485 (19)	0.18304 (10)	0.0553 (4)	
H10	−0.2307	−0.0165	0.1327	0.066*	
C11	−0.05416 (13)	−0.00683 (15)	0.20323 (8)	0.0416 (3)	
C12	−0.01820 (15)	0.13746 (17)	0.07846 (8)	0.0469 (4)	
H12A	−0.0812	0.0708	0.0458	0.052 (4)*	
H12B	0.0521	0.1375	0.0526	0.056 (4)*	
C13	−0.06934 (12)	0.29955 (15)	0.07513 (7)	0.0380 (3)	
C14	−0.08168 (16)	0.53187 (18)	0.13659 (10)	0.0568 (4)	
H14	−0.0619	0.5940	0.1835	0.068*	
C15	−0.15277 (16)	0.5944 (2)	0.06597 (11)	0.0610 (4)	
H15	−0.1801	0.6961	0.0650	0.073*	
C16	−0.18271 (15)	0.5037 (2)	−0.00321 (10)	0.0600 (4)	
H16	−0.2305	0.5428	−0.0524	0.072*	
C17	−0.14106 (13)	0.35385 (18)	0.00120 (9)	0.0496 (4)	
H17	−0.1608	0.2897	−0.0449	0.060*	
C18	0.48944 (16)	−0.2503 (2)	0.06534 (11)	0.0633 (4)	
H18A	0.4381	−0.3218	0.0286	0.095*	
H18B	0.5309	−0.3033	0.1147	0.095*	
H18C	0.5499	−0.2068	0.0386	0.095*	

### Atomic displacement parameters (Å^2^)

**Table:**

	*U*^11^	*U*^22^	*U*^33^	*U*^12^	*U*^13^	*U*^23^
O1	0.0632 (7)	0.0749 (8)	0.0368 (6)	0.0062 (6)	0.0106 (5)	0.0119 (5)
N1	0.0609 (8)	0.0670 (9)	0.0474 (8)	0.0091 (7)	0.0107 (6)	0.0143 (6)
N2	0.0487 (7)	0.0438 (7)	0.0433 (7)	0.0026 (5)	0.0050 (5)	0.0042 (5)
N3	0.0491 (7)	0.0372 (6)	0.0381 (6)	0.0050 (5)	0.0062 (5)	0.0019 (5)
N4	0.0572 (7)	0.0463 (7)	0.0355 (6)	0.0096 (6)	0.0050 (5)	−0.0026 (5)
C1	0.0454 (8)	0.0521 (9)	0.0390 (8)	−0.0074 (7)	0.0056 (6)	0.0096 (6)
C2	0.0527 (8)	0.0491 (8)	0.0355 (7)	−0.0054 (7)	0.0040 (6)	0.0085 (6)
C3	0.0440 (8)	0.0443 (8)	0.0414 (8)	−0.0110 (6)	0.0059 (6)	0.0065 (6)
C4	0.0578 (9)	0.0440 (8)	0.0526 (9)	−0.0097 (7)	0.0100 (7)	−0.0012 (7)
C5	0.0484 (8)	0.0321 (7)	0.0420 (8)	0.0014 (6)	0.0068 (6)	−0.0029 (6)
C6	0.0475 (8)	0.0373 (7)	0.0432 (8)	0.0054 (6)	0.0096 (6)	−0.0002 (6)
C7	0.0647 (10)	0.0542 (9)	0.0504 (9)	0.0019 (8)	0.0161 (8)	0.0061 (7)
C8	0.0718 (12)	0.0661 (11)	0.0664 (11)	−0.0091 (9)	0.0321 (9)	−0.0011 (9)
C9	0.0516 (10)	0.0804 (12)	0.0689 (11)	−0.0106 (9)	0.0216 (9)	−0.0147 (10)
C10	0.0481 (9)	0.0634 (10)	0.0520 (9)	0.0048 (7)	0.0063 (7)	−0.0097 (8)
C11	0.0475 (8)	0.0354 (7)	0.0419 (8)	0.0058 (6)	0.0100 (6)	−0.0044 (6)
C12	0.0575 (9)	0.0478 (8)	0.0337 (7)	0.0062 (7)	0.0068 (6)	−0.0024 (6)
C13	0.0392 (7)	0.0435 (8)	0.0306 (7)	0.0010 (6)	0.0067 (5)	0.0019 (6)
C14	0.0714 (11)	0.0463 (9)	0.0523 (9)	0.0088 (8)	0.0136 (8)	−0.0049 (7)
C15	0.0641 (10)	0.0475 (9)	0.0717 (12)	0.0137 (8)	0.0164 (9)	0.0134 (8)
C16	0.0548 (9)	0.0641 (10)	0.0551 (10)	0.0061 (8)	0.0001 (7)	0.0237 (8)
C17	0.0514 (9)	0.0563 (9)	0.0365 (8)	−0.0040 (7)	0.0004 (6)	0.0035 (7)
C18	0.0676 (11)	0.0635 (10)	0.0621 (10)	0.0020 (8)	0.0216 (9)	0.0026 (8)

### Geometric parameters (Å, °)

**Table:**

O1—C1	1.3526 (17)	C7—H7	0.9300
O1—N1	1.4100 (17)	C8—C9	1.388 (2)
N1—C3	1.3044 (17)	C8—H8	0.9300
N2—C5	1.3079 (17)	C9—C10	1.374 (2)
N2—C6	1.3880 (18)	C9—H9	0.9300
N3—C5	1.3720 (17)	C10—C11	1.390 (2)
N3—C11	1.3840 (18)	C10—H10	0.9300
N3—C12	1.4548 (16)	C12—C13	1.5090 (19)
N4—C13	1.3270 (16)	C12—H12A	0.9700
N4—C14	1.3414 (19)	C12—H12B	0.9700
C1—C2	1.338 (2)	C13—C17	1.3833 (18)
C1—C18	1.478 (2)	C14—C15	1.366 (2)
C2—C3	1.407 (2)	C14—H14	0.9300
C2—H2	0.9300	C15—C16	1.368 (2)
C3—C4	1.504 (2)	C15—H15	0.9300
C4—C5	1.494 (2)	C16—C17	1.372 (2)
C4—H4B	0.9700	C16—H16	0.9300
C4—H4A	0.9700	C17—H17	0.9300
C6—C7	1.390 (2)	C18—H18A	0.9600
C6—C11	1.3945 (19)	C18—H18B	0.9600
C7—C8	1.375 (2)	C18—H18C	0.9600
			
C1—O1—N1	108.57 (11)	C10—C9—H9	119.0
C3—N1—O1	105.28 (11)	C8—C9—H9	119.0
C5—N2—C6	104.75 (11)	C9—C10—C11	116.48 (15)
C5—N3—C11	106.49 (11)	C9—C10—H10	121.8
C5—N3—C12	127.86 (12)	C11—C10—H10	121.8
C11—N3—C12	125.63 (12)	N3—C11—C10	132.63 (13)
C13—N4—C14	116.77 (12)	N3—C11—C6	105.05 (12)
C2—C1—O1	109.15 (13)	C10—C11—C6	122.32 (14)
C2—C1—C18	134.95 (14)	N3—C12—C13	115.46 (11)
O1—C1—C18	115.90 (13)	N3—C12—H12A	108.4
C1—C2—C3	105.48 (12)	C13—C12—H12A	108.4
C1—C2—H2	127.3	N3—C12—H12B	108.4
C3—C2—H2	127.3	C13—C12—H12B	108.4
N1—C3—C2	111.51 (13)	H12A—C12—H12B	107.5
N1—C3—C4	119.88 (13)	N4—C13—C17	122.74 (13)
C2—C3—C4	128.61 (13)	N4—C13—C12	118.28 (11)
C5—C4—C3	112.85 (12)	C17—C13—C12	118.90 (12)
C5—C4—H4B	109.0	N4—C14—C15	124.12 (15)
C3—C4—H4B	109.0	N4—C14—H14	117.9
C5—C4—H4A	109.0	C15—C14—H14	117.9
C3—C4—H4A	109.0	C14—C15—C16	118.38 (15)
H4B—C4—H4A	107.8	C14—C15—H15	120.8
N2—C5—N3	113.28 (12)	C16—C15—H15	120.8
N2—C5—C4	124.10 (13)	C15—C16—C17	118.87 (14)
N3—C5—C4	122.62 (12)	C15—C16—H16	120.6
N2—C6—C7	129.63 (13)	C17—C16—H16	120.6
N2—C6—C11	110.42 (12)	C16—C17—C13	119.12 (14)
C7—C6—C11	119.95 (14)	C16—C17—H17	120.4
C8—C7—C6	117.89 (15)	C13—C17—H17	120.4
C8—C7—H7	121.1	C1—C18—H18A	109.5
C6—C7—H7	121.1	C1—C18—H18B	109.5
C7—C8—C9	121.43 (16)	H18A—C18—H18B	109.5
C7—C8—H8	119.3	C1—C18—H18C	109.5
C9—C8—H8	119.3	H18A—C18—H18C	109.5
C10—C9—C8	121.93 (16)	H18B—C18—H18C	109.5

### Hydrogen-bond geometry (Å, °)

**Table:**

*D*—H···*A*	*D*—H	H···*A*	*D*···*A*	*D*—H···*A*
C2—H2···N4^i^	0.93	2.52	3.385 (2)	155
C12—H12B···N1	0.97	2.60	3.479 (2)	151

Symmetry codes: (i) −*x*+1/2, *y*−1/2, −*z*+1/2.
